# 1,4-Bis(6-chloro­pyrimidin-4-yl­oxy)benzene

**DOI:** 10.1107/S1600536808043511

**Published:** 2009-01-08

**Authors:** Wen Ma, Hongyan Li

**Affiliations:** a College of Chemistry and Chemical Engineering, Xinyang Normal Universty, Xinyang, Henan 464000, People’s Republic of China; b College of Life Science, Xinyang Normal Universty, Xinyang, Henan 464000, People’s Republic of China

## Abstract

In the title compound, C_14_H_8_Cl_2_N_4_O_2_, all atoms of the 6-chloro­pyrimidin-4-yl­oxy group and the C atoms at the *para* positions of the central benzene ring lie on a crystallographic mirror plane. The complete benzene ring is generated by the mirror plane and hence the dihedral angles between the pyrimidine rings and the benzene ring are exactly 90°. The crystal structure is stabilized by weak C—H⋯O and C—H⋯N hydrogen bonds.

## Related literature

For background information, see: Halim *et al.* (1999 ▶); Meng & Huang (2000 ▶); Maes *et al.* (2003 ▶); Friend *et al.* (1999 ▶).
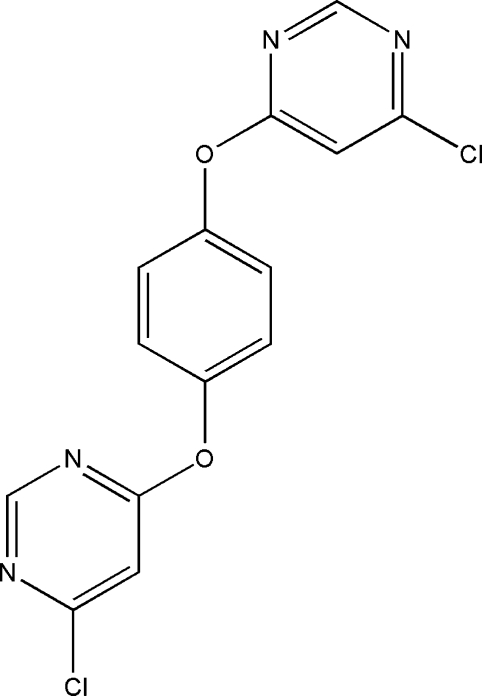


## Experimental

### 

#### Crystal data

C_14_H_8_Cl_2_N_4_O_2_
*M* *_r_* = 335.14
Monoclinic, 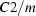

*a* = 19.0760 (5) Å
*b* = 6.9693 (2) Å
*c* = 10.7893 (3) Å
β = 93.301 (3)°
*V* = 1432.02 (7) Å^3^
*Z* = 4
Mo *K*α radiation
μ = 0.47 mm^−1^
*T* = 298 (2) K
0.20 × 0.10 × 0.10 mm

#### Data collection

Bruker SMART CCD diffractometer
Absorption correction: none
6918 measured reflections
1372 independent reflections
1156 reflections with *I* > 2σ(*I*)’
*R*_int_ = 0.050

#### Refinement

*R*[*F* ^2^ > 2σ(*F* ^2^)] = 0.037
*wR*(*F* ^2^) = 0.105
*S* = 1.07
1372 reflections
128 parameters
H-atom parameters constrained
Δρ_max_ = 0.22 e Å^−3^
Δρ_min_ = −0.25 e Å^−3^

### 

Data collection: *SMART* (Bruker, 2007 ▶); cell refinement: *SAINT* (Bruker, 2007 ▶); data reduction: *SAINT*; program(s) used to solve structure: *SHELXS97* (Sheldrick, 2008 ▶); program(s) used to refine structure: *SHELXL97* (Sheldrick, 2008 ▶); molecular graphics: *SHELXTL* (Sheldrick, 2008 ▶); software used to prepare material for publication: *PLATON* (Spek, 2003 ▶).

## Supplementary Material

Crystal structure: contains datablocks global, I. DOI: 10.1107/S1600536808043511/lh2746sup1.cif

Structure factors: contains datablocks I. DOI: 10.1107/S1600536808043511/lh2746Isup2.hkl

Additional supplementary materials:  crystallographic information; 3D view; checkCIF report

## Figures and Tables

**Table 1:** Hydrogen-bond geometry (Å, °)

*D*—H⋯*A*	*D*—H	H⋯*A*	*D*⋯*A*	*D*—H⋯*A*
C10—H10⋯O2^i^	0.93	2.57	3.463 (3)	161
C3—H3⋯N2^ii^	0.93	2.48	3.355 (3)	157

Symmetry codes: (i) 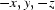; (ii) 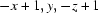.

**Fig. 1.**
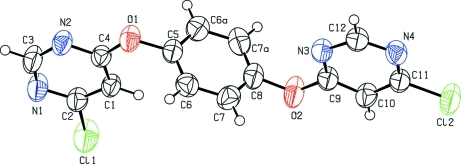
The molecular structure of (I), showing the atom-numbering scheme. Displacement ellipsoids are drawn at the 20% probability level [Symmetry codes: (a) x,-y,z].

**Fig. 2.**
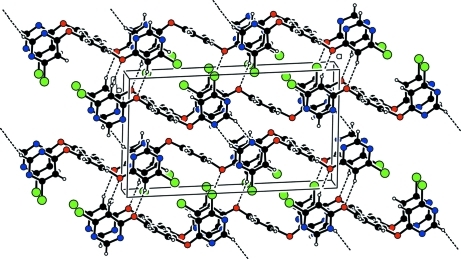
Part of the crystal structure with hydrogen bonds shown by dashed lines.

## supplementary crystallographic information

### Comment

In recent publications it has been shown that polymers consisting of heterocyclic building blocks can be used in organic light-emitting diodes (LEDs) because of their electroluminescent properties. (Halim *et al.*, 1999; Friend *et al.*,1999; Meng & Huang, 2000; Maes *et al.*, 2003). We report here the synthesis and crystal structure the title compound (I) (Fig. 1). All atoms of the 6-chloropyrimidin-4-yloxy group and the C atoms at the para positions of the central benzene ring lie on a crystallographic mirror plane. The symmetry complete benzene ring is generated by the mirror plane and hence the dihedral angles between the pyrimidine rings and the benzene ring are exactly 90°.  The crystal packing is stabilized by weak intermolecular hydrogen bonds interactions. Each molecule acts as a donor and acceptor to form C–H···O and C–H···N hydrogen bonds with two other symmetry related molecules, forming a chain run parallel to [101] (Fig. 2; Table 2).

### Experimental

Hydroquinone 0.55 g(5 mmol) was dissolved in 50 ml CH_3_CN, the solution was stirred at room temperature for 0.5 h with an excess of anhydrous K_2_CO_3_(2.5 equiv.). After another 0.5 h of reflux, 4,6-dichloropyrimidine2.59 g (10 mmol) in 20 ml CH_3_CN was added and the mixture was refluxed for 4 h. After evaporation of the solvent, water was added and the mixture was extracted with CH_2_Cl_2_ and dried over MgSO_4_. The products were purified by column chromatography (hexanes/ethyl acetate, 5:1) and obtained as white solids. Colourless block-shapped crystals were obtained by evaporation of CH_2_Cl_2_.

### Refinement

After being located in the difference map, all H-atoms were fixed geometrically at ideal positions and allowed to ride on the parent C atoms with C—H = 0.93Å and *U*_iso_(H)= 1.2*U*_eq_.

### Crystal data

**Table:**

C_14_H_8_Cl_2_N_4_O_2_	*F*(000) = 680
*M**_r_* = 335.14	*D*_x_ = 1.555 Mg m^−^^3^
Monoclinic, *C*2/*m*	Mo *K*α radiation, λ = 0.71073 Å
Hall symbol: -C 2y	Cell parameters from 3581 reflections
*a* = 19.0760 (5) Å	θ = 2.8–27.8°
*b* = 6.9693 (2) Å	µ = 0.47 mm^−^^1^
*c* = 10.7893 (3) Å	*T* = 298 K
β = 93.301 (3)°	Block, colorless
*V* = 1432.02 (7) Å^3^	0.20 × 0.10 × 0.10 mm
*Z* = 4	

### Data collection

**Table:**

Bruker SMART CCD diffractometer	1156 reflections with *I* > 2σ(*I*)'
Radiation source: fine-focus sealed tube	*R*_int_ = 0.050
graphite	θ_max_ = 25.0°, θ_min_ = 1.9°
φ and ω scans	*h* = −22→22
6918 measured reflections	*k* = −8→7
1372 independent reflections	*l* = −12→12

### Refinement

**Table:**

Refinement on *F*^2^	Secondary atom site location: difference Fourier map
Least-squares matrix: full	Hydrogen site location: inferred from neighbouring sites
*R*[*F*^2^ > 2σ(*F*^2^)] = 0.037	H-atom parameters constrained
*wR*(*F*^2^) = 0.105	*w* = 1/[σ^2^(*F*_o_^2^) + (0.0635*P*)^2^ + 0.2906*P*] where *P* = (*F*_o_^2^ + 2*F*_c_^2^)/3
*S* = 1.07	(Δ/σ)_max_ < 0.001
1372 reflections	Δρ_max_ = 0.22 e Å^−^^3^
128 parameters	Δρ_min_ = −0.25 e Å^−^^3^
0 restraints	Extinction correction: *SHELXL97* (Sheldrick, 2008), Fc^*^=kFc[1+0.001xFc^2^λ^3^/sin(2θ)]^-1/4^
Primary atom site location: structure-invariant direct methods	Extinction coefficient: 0.0062 (13)

### Special details

**Table:**

Geometry. All e.s.d.'s (except the e.s.d. in the dihedral angle between two l.s. planes) are estimated using the full covariance matrix. The cell e.s.d.'s are taken into account individually in the estimation of e.s.d.'s in distances, angles and torsion angles; correlations between e.s.d.'s in cell parameters are only used when they are defined by crystal symmetry. An approximate (isotropic) treatment of cell e.s.d.'s is used for estimating e.s.d.'s involving l.s. planes.
Refinement. Refinement of *F*^2^ against ALL reflections. The weighted *R*-factor *wR* and goodness of fit *S* are based on *F*^2^, conventional *R*-factors *R* are based on *F*, with *F* set to zero for negative *F*^2^. The threshold expression of *F*^2^ > σ(*F*^2^) is used only for calculating *R*-factors(gt) *etc*. and is not relevant to the choice of reflections for refinement. *R*-factors based on *F*^2^ are statistically about twice as large as those based on *F*, and *R*- factors based on ALL data will be even larger.

### Fractional atomic coordinates and isotropic or equivalent isotropic displacement parameters (Å^2^)

**Table:**

	*x*	*y*	*z*	*U*_iso_*/*U*_eq_	
C1	0.34316 (10)	0.0000	0.22381 (18)	0.0495 (5)	
H1	0.3002	0.0000	0.1786	0.059*	
C2	0.40565 (11)	0.0000	0.16857 (19)	0.0543 (6)	
C3	0.46576 (12)	0.0000	0.3504 (2)	0.0666 (7)	
H3	0.5089	0.0000	0.3950	0.080*	
C4	0.34857 (11)	0.0000	0.35261 (19)	0.0467 (5)	
C5	0.22554 (11)	0.0000	0.36269 (19)	0.0512 (6)	
C6	0.19419 (8)	0.1719 (3)	0.33512 (14)	0.0589 (4)	
H6	0.2162	0.2867	0.3578	0.071*	
C7	0.12865 (9)	0.1712 (3)	0.27244 (15)	0.0636 (5)	
H7	0.1058	0.2859	0.2523	0.076*	
C8	0.09825 (11)	0.0000	0.24084 (19)	0.0581 (7)	
C9	−0.02669 (11)	0.0000	0.21792 (19)	0.0529 (6)	
C10	−0.08588 (12)	0.0000	0.1385 (2)	0.0570 (6)	
H10	−0.0839	0.0000	0.0526	0.068*	
C11	−0.14794 (11)	0.0000	0.1964 (2)	0.0525 (5)	
C12	−0.09188 (11)	0.0000	0.3830 (2)	0.0553 (6)	
H12	−0.0939	0.0000	0.4689	0.066*	
Cl1	0.40498 (4)	0.0000	0.00858 (5)	0.0916 (3)	
Cl2	−0.22616 (3)	0.0000	0.10747 (6)	0.0829 (3)	
N1	0.46804 (10)	0.0000	0.22748 (18)	0.0676 (6)	
N2	0.40951 (9)	0.0000	0.41739 (17)	0.0572 (5)	
N3	−0.02774 (9)	0.0000	0.33990 (16)	0.0533 (5)	
N4	−0.15287 (9)	0.0000	0.31826 (17)	0.0566 (5)	
O1	0.29285 (8)	0.0000	0.42430 (14)	0.0619 (5)	
O2	0.03558 (8)	0.0000	0.16482 (14)	0.0793 (6)	

### Atomic displacement parameters (Å^2^)

**Table:**

	*U*^11^	*U*^22^	*U*^33^	*U*^12^	*U*^13^	*U*^23^
C1	0.0393 (11)	0.0687 (14)	0.0399 (11)	0.000	−0.0027 (9)	0.000
C2	0.0453 (12)	0.0777 (15)	0.0400 (11)	0.000	0.0022 (9)	0.000
C3	0.0407 (12)	0.109 (2)	0.0495 (13)	0.000	−0.0062 (10)	0.000
C4	0.0421 (11)	0.0573 (12)	0.0403 (10)	0.000	0.0002 (8)	0.000
C5	0.0397 (11)	0.0797 (16)	0.0345 (10)	0.000	0.0054 (8)	0.000
C6	0.0543 (9)	0.0708 (11)	0.0520 (9)	−0.0059 (8)	0.0062 (7)	0.0019 (8)
C7	0.0528 (9)	0.0824 (12)	0.0561 (9)	0.0116 (9)	0.0073 (7)	0.0109 (9)
C8	0.0390 (11)	0.102 (2)	0.0340 (10)	0.000	0.0072 (8)	0.000
C9	0.0434 (11)	0.0779 (15)	0.0378 (11)	0.000	0.0067 (9)	0.000
C10	0.0484 (12)	0.0836 (16)	0.0389 (11)	0.000	0.0011 (9)	0.000
C11	0.0440 (11)	0.0612 (14)	0.0519 (12)	0.000	−0.0009 (9)	0.000
C12	0.0515 (13)	0.0762 (16)	0.0389 (11)	0.000	0.0078 (9)	0.000
Cl1	0.0605 (4)	0.1762 (9)	0.0387 (4)	0.000	0.0072 (3)	0.000
Cl2	0.0467 (4)	0.1308 (7)	0.0695 (5)	0.000	−0.0106 (3)	0.000
N1	0.0415 (10)	0.1109 (17)	0.0503 (11)	0.000	0.0013 (9)	0.000
N2	0.0441 (10)	0.0836 (14)	0.0429 (9)	0.000	−0.0057 (8)	0.000
N3	0.0452 (10)	0.0774 (13)	0.0375 (9)	0.000	0.0050 (8)	0.000
N4	0.0447 (10)	0.0750 (13)	0.0509 (11)	0.000	0.0095 (8)	0.000
O1	0.0430 (8)	0.1051 (13)	0.0376 (7)	0.000	0.0019 (6)	0.000
O2	0.0403 (9)	0.1598 (19)	0.0379 (8)	0.000	0.0040 (7)	0.000

### Geometric parameters (Å, °)

**Table:**

C1—C2	1.363 (3)	C7—C8	1.361 (2)
C1—C4	1.388 (3)	C7—H7	0.9300
C1—H1	0.9300	C8—C7^i^	1.361 (2)
C2—N1	1.317 (3)	C8—O2	1.410 (3)
C2—Cl1	1.725 (2)	C9—N3	1.317 (3)
C3—N2	1.328 (3)	C9—O2	1.348 (3)
C3—N1	1.329 (3)	C9—C10	1.377 (3)
C3—H3	0.9300	C10—C11	1.370 (3)
C4—N2	1.322 (3)	C10—H10	0.9300
C4—O1	1.350 (2)	C11—N4	1.323 (3)
C5—C6^i^	1.364 (2)	C11—Cl2	1.727 (2)
C5—C6	1.364 (2)	C12—N4	1.322 (3)
C5—O1	1.411 (3)	C12—N3	1.334 (3)
C6—C7	1.387 (2)	C12—H12	0.9300
C6—H6	0.9300		
			
C2—C1—C4	114.92 (19)	C7^i^—C8—C7	122.4 (2)
C2—C1—H1	122.5	C7^i^—C8—O2	118.70 (11)
C4—C1—H1	122.5	C7—C8—O2	118.70 (11)
N1—C2—C1	125.3 (2)	N3—C9—O2	119.27 (19)
N1—C2—Cl1	115.94 (16)	N3—C9—C10	124.21 (19)
C1—C2—Cl1	118.76 (17)	O2—C9—C10	116.52 (19)
N2—C3—N1	128.1 (2)	C11—C10—C9	114.6 (2)
N2—C3—H3	115.9	C11—C10—H10	122.7
N1—C3—H3	115.9	C9—C10—H10	122.7
N2—C4—O1	113.24 (18)	N4—C11—C10	124.4 (2)
N2—C4—C1	122.83 (19)	N4—C11—Cl2	116.32 (17)
O1—C4—C1	123.93 (19)	C10—C11—Cl2	119.24 (18)
C6^i^—C5—C6	122.8 (2)	N4—C12—N3	127.8 (2)
C6^i^—C5—O1	118.57 (10)	N4—C12—H12	116.1
C6—C5—O1	118.57 (10)	N3—C12—H12	116.1
C5—C6—C7	118.36 (17)	C2—N1—C3	113.6 (2)
C5—C6—H6	120.8	C4—N2—C3	115.20 (19)
C7—C6—H6	120.8	C9—N3—C12	114.53 (19)
C8—C7—C6	118.97 (17)	C12—N4—C11	114.45 (18)
C8—C7—H7	120.5	C4—O1—C5	117.07 (16)
C6—C7—H7	120.5	C9—O2—C8	119.40 (16)
			
C4—C1—C2—N1	0.0	C1—C4—N2—C3	0.0
C4—C1—C2—Cl1	180.0	N1—C3—N2—C4	0.0
C2—C1—C4—N2	0.0	O2—C9—N3—C12	180.0
C2—C1—C4—O1	180.0	C10—C9—N3—C12	0.0
C6^i^—C5—C6—C7	−2.3 (3)	N4—C12—N3—C9	0.0
O1—C5—C6—C7	178.76 (14)	N3—C12—N4—C11	0.0
C5—C6—C7—C8	−0.1 (3)	C10—C11—N4—C12	0.0
C6—C7—C8—C7^i^	2.4 (3)	Cl2—C11—N4—C12	180.0
C6—C7—C8—O2	−172.75 (14)	N2—C4—O1—C5	180.0
N3—C9—C10—C11	0.0	C1—C4—O1—C5	0.0
O2—C9—C10—C11	180.0	C6^i^—C5—O1—C4	90.53 (16)
C9—C10—C11—N4	0.0	C6—C5—O1—C4	−90.53 (16)
C9—C10—C11—Cl2	180.0	N3—C9—O2—C8	0.0
C1—C2—N1—C3	0.0	C10—C9—O2—C8	180.0
Cl1—C2—N1—C3	180.0	C7^i^—C8—O2—C9	92.31 (16)
N2—C3—N1—C2	0.0	C7—C8—O2—C9	−92.31 (16)
O1—C4—N2—C3	180.0		

Symmetry codes: (i) *x*, −*y*, *z*.

### Hydrogen-bond geometry (Å, °)

**Table:**

*D*—H···*A*	*D*—H	H···*A*	*D*···*A*	*D*—H···*A*
C10—H10···O2^ii^	0.93	2.57	3.463 (3)	161
C3—H3···N2^iii^	0.93	2.48	3.355 (3)	157

Symmetry codes: (ii) −*x*, *y*, −*z*; (iii) −*x*+1, *y*, −*z*+1.
